# Negative pressure characteristics of an evaporating meniscus at nanoscale

**DOI:** 10.1186/1556-276X-6-72

**Published:** 2011-01-12

**Authors:** Shalabh C Maroo, JN Chung

**Affiliations:** 1 Department of Mechanical and Aerospace Engineering, University of Florida, Gainesville, FL 32611, USA; 2 Department of Mechanical Engineering, M.I.T., Cambridge, MA 02139, USA

## Abstract

This study aims at understanding the characteristics of negative liquid pressures at the nanoscale using molecular dynamics simulation. A nano-meniscus is formed by placing liquid argon on a platinum wall between two nano-channels filled with the same liquid. Evaporation is simulated in the meniscus by increasing the temperature of the platinum wall for two different cases. Non-evaporating films are obtained at the center of the meniscus. The liquid film in the non-evaporating and adjacent regions is found to be under high absolute negative pressures. Cavitation cannot occur in these regions as the capillary height is smaller than the critical cavitation radius. Factors which determine the critical film thickness for rupture are discussed. Thus, high negative liquid pressures can be stable at the nanoscale, and utilized to create passive pumping devices as well as significantly enhance heat transfer rates.

## Introduction

The physical attributes of phenomenon associated with the nanoscale are different from those at the macroscale due to the length-scale effects. In nature, transport processes involving a meniscus are usually associated with nano- and micro-scales. Capillary forces are of main importance in micro- and macro-scale fluidic systems. However at nanoscale, disjoining forces can become extremely dominant. These disjoining forces can cause liquid films to be under high absolute negative pressures. A better insight into negative liquid pressures can be gained from the phase diagram of water, which shows the stable, metastable, and unstable regions [1]. Usually in such cases cavitation is observed, i.e., vapor bubbles form when a liquid is stretched. However, for the formation of a spherical vapor bubble, a critical radius of cavitation *R*_c _(defined as [2]: $R_{\text{c}}=-\frac{2\gamma_{\text{LV}}}{P_{\text{liquid}}}$) has to be achieved. Thus, if the radius of a bubble is greater than *R*_c_, it will grow unrestricted. No cavitation will occur if any dimension of the liquid film is smaller than *R*_c_, and the liquid can exist in a metastable state.

Briggs, in 1955, heated water in a thin-walled capillary tube, open to atmosphere, up to 267°C for about 5 s before explosion occurred, and concluded that during the short time before explosion occurs, the water must be under an internal negative pressure [3]. It has only been recently shown, through experiments that water can exist at extreme metastable states at the nanoscale. Water plugs at negative pressures of 17 ± 10 bar were achieved by filling water in a hydrophilic silicon oxide nano-channel of approximate height of 100 nm [4]. The force contribution in water capillary bridges formed between a nanoscale atomic force microscope tip and a silicon wafer sample was measured, and negative pressures down to -160 MPa were obtained [5]. Important consequences of the negative liquid pressures include the ascent of sap in tall trees [6], achieving boiling at temperatures much lower than saturation temperatures at corresponding vapor pressure [7], and liquid flow from bulk to evaporating film regions during heterogeneous bubble growth [8,9].

Molecular dynamics is a vital tool to simulate and characterize the importance of disjoining force effects on the existence of negative pressures in liquids at the nanoscale. It can also provide means to compare the strength of disjoining and capillary forces at such small scales, which has not yet been possible via experiments. Although negative liquid pressure has been experimentally shown for water, it should theoretically exist in other liquids as well. With this aim, we simulated two cases of nanoscale meniscus evaporation of liquid argon on platinum wall using molecular dynamics simulation. To the best of our knowledge, this is the first study to show the existence of negative liquid pressures via molecular simulations.

**Figure 1:**
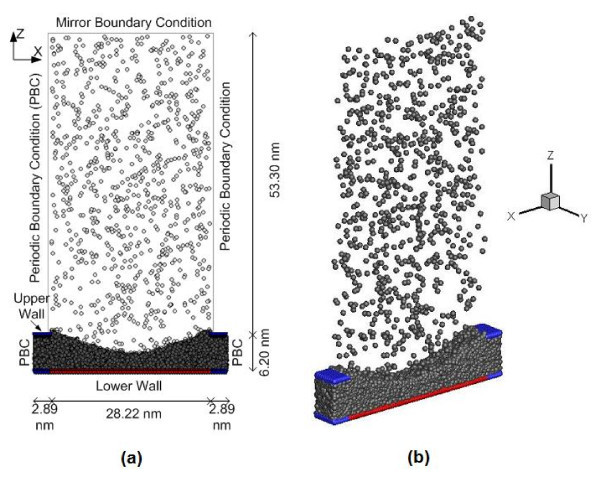
**Liquid argon meniscus, surrounded by argon vapor, in an opening constructed of platinum wall atoms**. **(a) **2D view along the *x*-*z *plane depicting the boundary conditions and dimensions, and **(b) **3D view of the simulation domain where the liquid-vapor interface can be clearly noticeable. Heat is transferred to the meniscus from the platinum wall region shown in red, while the region shown in blue is maintained at the lower initial temperature.

A meniscus is formed by placing liquid argon between a lower wall and an upper wall, with an opening in the upper wall as shown in Figure 1a, b. The walls are made of three layers of platinum (Pt) atoms arranged in fcc (111) structure. The space above the meniscus is occupied by argon vapor. The domain consists of a total of 14,172 argon atoms and 7,776 platinum atoms. The initial equilibrium temperature is 90 K. The time step is 5 fs. The atomic interaction is governed by the modified Lennard-Jones potential defined as [10]:

(1) $$U_{\text{MLJ}}(r)=4\varepsilon \left[\left\{{\left(\frac{\sigma }{r}\right)}^{12}-{\left(\frac{\sigma }{r}\right)}^{6}\right\}+\left\{6{\left(\frac{\sigma }{r_{\text{cut}}}\right)}^{12}-3{\left(\frac{\sigma }{r_{\text{cut}}}\right)}^{6}\right\}{\left(\frac{r}{r_{\text{cut}}}\right)}^{2}-\left\{7{\left(\frac{\sigma }{r_{\text{cut}}}\right)}^{12}-4{\left(\frac{\sigma }{r_{\text{cut}}}\right)}^{6}\right\}\right]$$

The above potential form is employed for both Ar-Ar and Ar-Pt interaction with the following values: *σ*_Ar-Ar _= 3.4 × 10^-10 ^m, *ε*_Ar-Ar _= 1.67 × 10^-21^ J, *σ*_Ar-Pt _= 3.085 × 10^-10 ^m, *ε*_Ar-Pt _= 0.894 × 10^-21 ^J.

The cutoff radius is set as *r*_cut _= 4*σ*_Ar-Ar _The force of interaction is calculated from the potential function by $\vec{F}=-\nabla U.$

All the boundaries in *x *and *y *directions are periodic. The width of the periodic boundary above the upper walls in the *x*-direction is restricted to the width of the opening. Any argon atom which goes above the upper walls does not interact with the wall atoms anymore. The top boundary in the *z*-direction is the mirror boundary condition where the argon atom is reflected back in the domain without any loss of energy, i.e., the boundary is adiabatic and elastic in nature. The 'fluid-wall thermal equilibrium model' is used to numerically simulate heat transfer between wall and fluid atoms [11,12]. The algorithm used to calculate the atomic force interactions is the linked-cell algorithm. The equations of motion are integrated in order to obtain the positions and velocities of the atoms at every time step. The integrator method used here is the Velocity-Verlet method. Liquid atoms are distinguished from vapor atoms based on the minimum number of neighboring atoms within a certain radius [11]. Vapor pressure is evaluated as defined elsewhere [13], which has been previously verified by the authors [14]. The simulation process is divided into three parts: velocity-scaling period, equilibration period, and the heating period. During the velocity-scaling period (0-500 ps), the velocity of each argon atom is scaled at every time step so that the system temperature remains constant. This is followed by the equilibration period (500-1000 ps) in which the velocity-scaling is removed and the argon atoms are allowed to move freely and equilibrate. The wall temperatures during these two steps are the same as the initial system temperature. At the start of the heating period (1000-3000 ps), heat is transferred to the meniscus from the platinum wall region and evaporation is observed. Two cases are simulated in this study:

### Case I

After the equilibrium period, the temperature of platinum wall underneath the opening (shown in red color in Figure 1) is simulated to be 130 K while the rest of the wall (shown in blue color in Figure 1) is kept at the initial temperature of 90 K.

### Case II

After the equilibrium period, temperatures of all walls are simulated to 130 K.

When a liquid film is thin enough, the liquid-vapor and liquid-solid interfaces interact with each other giving rise to disjoining pressure. Attractive forces from the solid act to pull the liquid molecules causing the liquid film to be at a lower pressure than the surrounding vapor pressure. A novel method to evaluate the disjoining forces for nanoscale thin films from molecular dynamics simulations has been introduced in a prior study [11]. Starting from the Lennard-Jones potential, which is the model of interaction between Ar and Pt, the following equation is derived:

(2) $$U_{\text{LJ}}(z)=-\frac{A}{12\pi }\left[\frac{1}{d^{2}}-\frac{1}{{\left(z\right)}^{2}}-\frac{\sigma_{\text{Ar-Pt}}^{6}}{30d^{8}}+\frac{\sigma_{\text{Ar-Pt}}^{6}}{30{\left(z\right)}^{8}}\right]$$

where *A *is the Hamaker constant, *d *is the gap between Ar and Pt slabs, *z *is the total thickness of the Ar slab (including the gap thickness), and *U*_LJ _is the total interaction energy between Ar and Pt slabs from molecular dynamics using LJ potential. This equation was used to evaluate the Hamaker constant for the non-evaporating argon film with varying pressure and temperature [14], and an average value of *A *= 6.13 × 10^-20 ^J is used in this study. The disjoining pressure, for non-polar molecules, is calculated as:

(3) $$P_{\text{d}}=-\frac{dU_{\text{LJ}}(z)}{dz}=\frac{A}{12\pi }\left[\frac{2}{{\left(z\right)}^{3}}-\frac{8\sigma_{\text{Ar-Pt}}^{6}}{30{\left(z\right)}^{9}}\right]$$

From the classical capillary equation, the capillary pressure is the product of interfacial curvature *K *and surface tension coefficient σ as follows:

(4) $$\begin{array}{cc} P_{\text{c}}=\sigma K, & K=\delta^{\prime \prime }{\left(1+{\delta^{\prime }}^{2}\right)}^{-1.5} \end{array}$$

where *δ' *and *δ" *are, respectively, the first and second derivatives of film thickness with respect to *x*-position. Equation 4, although a macroscopic formula, serves as a good approximation [15]. The variation of meniscus thickness is determined in the *x*-*z *plane at different time intervals. The meniscus, formed from liquid argon atoms, is divided into square bins of dimension 1*σ*_Ar-Ar _× 1*σ*_Ar-Ar _and the number of atoms in each square is determined. A check is performed from the Pt wall in the positive *z*-direction such that if the number of atoms in a bin falls below 0.5 times the average number density, an interface marker is placed at the center of that bin. Interface markers are placed to determine the meniscus interface using this procedure, and a fourth-order polynomial fit of these markers is used to obtain the function *δ*(*x*).

**Figure 2:**
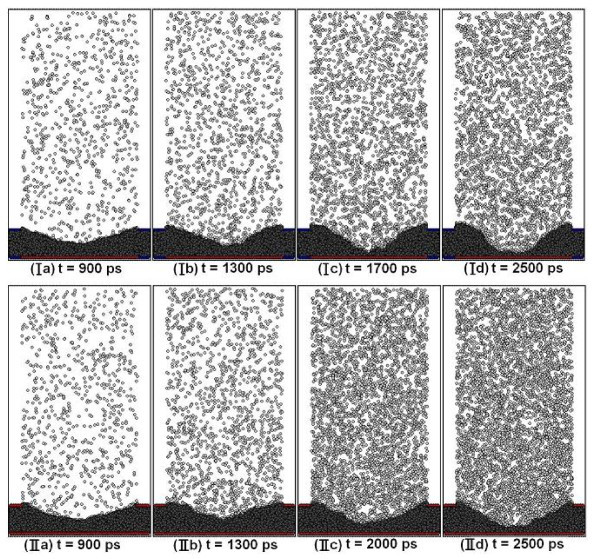
***X*-*Z *plane of simulation domain at different time intervals for Case I and Case II**. Evaporation of the liquid meniscus is seen, with the formation of the non-evaporating film at the center of the meniscus toward the end of the simulation period at *t *= 2500 ps for both cases.

Figure 2 shows the snapshots of the computational domain at different time intervals for Case I and Case II. For Case I, as shown in Figure 2-Ia, the liquid-vapor interface of the meniscus is clearly noticeable as evaporation has not yet started and surface tension assists in the formation of the interface. Vigorous evaporation is seen in Figure 2-Ib which results in an uneven meniscus interface. Evaporation rate slows down with time due to: (i) an increase in pressure in the gas phase, (ii) majority of liquid atoms at the center of the meniscus have evaporated, and (iii) liquid meniscus near the nano-channels is cooler than the vapor temperature causing condensation at the meniscus edges in Case I. With continuous evaporation taking place, the thinnest part of the meniscus at the center continues to decrease in thickness until a uniform non-evaporating film forms (Figure 2-Id). Unlike Case I, since all walls are at a higher temperature and liquid argon in the nano-channels is also heated up, Case II results in higher evaporation flux and increased mobility of atoms. Hence, as can be seen from Figure 2-IId, the non-evaporating film thickness is greater and the meniscus is less steep in curvature compared to Case I.

**Figure 3:**
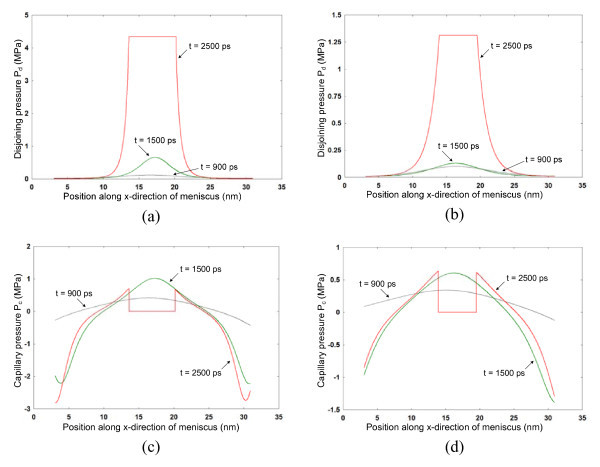
**Disjoining pressure variation in the liquid meniscus for **(a) **Case I, and **(b) **Case II, and capillary pressure variation in the liquid meniscus for **(c) **Case I, and **(d) **Case II**. Pressure variations are shown at three time intervals of *t *= 900, 1500, and 2500 ps. Disjoining forces can be significantly dominant for ultra-thin films at nanoscale compared to capillary forces.

Figure 3a, b shows the disjoining pressure variation along the width of the meniscus at three different time steps for Case I and Case II, respectively. Disjoining pressure increases significantly upon the formation of the non-evaporating film. The disjoining pressure is greater for Case I (*P*_d _= 4.34 MPa) than Case II (*P*_d _= 1.31 MPa) as expected. Due to higher temperature throughout the meniscus in Case II, the atoms have higher freedom to rearrange in a more uniform curvature resulting in an increase in film thickness of the non-evaporating film at the center of the meniscus compared to Case I.

The disjoining pressure quickly goes down to near-zero values as the meniscus thickness increases away from the non-evaporating film region. The capillary pressure variation is shown in Figure 3c, d for Case I and Case II, respectively. The capillary pressure is zero in the non-evaporating region as the non-evaporating film has a flat interface. A capillary pressure gradient exists in the meniscus region. Capillary pressure reaches negative values at the edge of the meniscus due to curvature effects and is a result of the simulation domain studied here. Comparing the disjoining and capillary pressure values, it is seen that disjoining forces dominate in nanoscale ultra-thin films, as related by Equation 3, while capillary forces become prominent with increase in film thickness and curvature.

**Figure 4:**
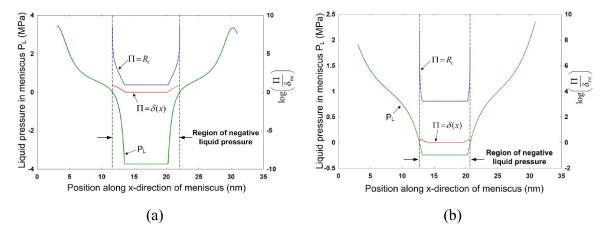
**Variation in liquid pressure along the meniscus at *t *= 2500 ps for **(a) **Case I, and **(b) **Case II**. High negative pressure values are seen at the center of the meniscus. A normalized function log(Π/*δ*_ne_) is plotted in the region of negative liquid pressure for Π = *R*_c _= -2*γ*/*P*_L _and Π = *δ*(*x*), which nullifies the possibility of cavitation in this region as the meniscus thickness is smaller than the critical cavitation radius.

The pressure in the liquid film is obtained using the augmented Young-Laplace equation: *P*_L _= *P*_v _- *P*_c _- *P*_d_, where *P*_L _is the liquid pressure and *P*_v _is the vapor pressure. The average vapor pressure values at *t *= 2500 ps for Case I and Case II are 0.613 and 1.071 MPa, respectively. Figure 4a, b depicts the variation in liquid pressure along the meniscus for Case I and Case II, respectively. Due to high disjoining pressure in the non-evaporation film region, and partially due to capillary forces in its adjacent regions, the liquid is found to be under high negative pressure at the center of the meniscus. Usually, at macroscale, liquid regions subject to negative pressures cavitate. However, at nanoscale, cavitation can be avoided if the critical cavitation radius is larger than the smallest characteristic dimension [16]. To verify this aspect in our study, a normalized function log(Π/*δ*_ne_) is plotted in the region of negative liquid pressure for Π = *R*_c _= -2*γ*/*P*_L _and Π = *δ*(*x*), as shown in Figure 4a, b, where *δ*_ne _is the thickness of the non-evaporating film. The normalized function has higher values for Π = *R*_c _compared to Π = *δ*(*x*), which signifies that the critical cavitation radius is larger than the meniscus height. Thus, the liquid meniscus region under high negative pressures can exist in a metastable state.

Figure 4 also provides insight into the factors which determine the stability of such films. The difference between the normalized function values for Π = *R*_c _and Π = *δ*(*x*) is smaller for Case I than Case II, which implies that the tendency for the liquid film to rupture is higher for Case I. The following question arises: what is the critical thickness *δ*_cr _at which these liquid films would rupture, i.e., cavitate? This can be determined from the definitions of critical cavitation radius and augmented Young-Laplace equation, i.e., *δ*_cr _= -2*γ*/[*P*_v _- *P*_c_(*δ*_cr_) - *P*_d_(*δ*_cr_)]. In the case of non-evaporating films, which form during heterogeneous bubble growth, this equation can be simplified by assuming *P*_c _= 0 due to the planar nature of the film. Using Equation 3 where the repulsive term can be neglected as *σ *for liquid-solid interaction is generally smaller than *δ *by an order of magnitude, the following equation can be derived: 6*πP*_v_*δ*^3^_cr _+ 12*πγδ*^2^_cr _*A *= 0, which can be solved analytically to determine the critical thickness for rupture. It can be seen that *δ*_cr _is a function of the vapor pressure, substrate temperature (indirectly via the liquid-vapor surface tension term), and substrate-liquid interaction (embedded in the Hamaker constant *A*). Premature rupture of non-evaporating film during bubble growth can lead to significant increase in pool boiling heat transfer and delaying the critical heat flux limit.

Negative pressure in liquids has been a point of interest over past several decades. An attempt has been made in this work to study and quantify the components of negative pressures in evaporating nano-menisci using molecular dynamics simulation. The disjoining and capillary pressures are evaluated in an evaporating meniscus at the nanoscale. Disjoining forces significantly dominate the capillary forces for ultra-thin films at the nanoscale. Liquid pressure in the meniscus is calculated using the augmented Young-Laplace equation. The center of the meniscus is found to be under high absolute negative pressures. It is shown that cavitation cannot occur as the critical cavitation radius is larger than the thickness of the meniscus. The factors determining the critical film thickness required for rupture are discussed. This property of sustaining high negative pressures at the nanoscale can be engineered to provide passive transport of liquid, and applied in power devices to attain significantly higher heat rejection rates, which is one of the major bottlenecks in achieving next generation computer chips, nuclear reactors, and rocket engines. This study serves as a first step toward understanding pressure characteristics in capillaries at the nanoscale using molecular simulations, with water nano-capillaries being the most intriguing and a near future goal.

## Competing interests

The authors declare that they have no competing interests.

## Authors' contributions

SCM participated in conceiving the study, wrote the simulation code, carried out the simulations and results analysis, and drafted the manuscript. JNC participated in conceiving the study, advised in results analysis and helped to draft the manuscript. All authors read and approved the final manuscript.
